# Supplementary material for: Impacts of mutation accumulation and order on tumor initiation revealed by engineered murine colorectal cancer organoids
Source: eLife. 2026 Nov 3;15:RP110070. doi: 10.7554/eLife.110070 (PMC13730527; doi:10.7554/eLife.110070)
Supplement: Supplementary file 1 [file elife-110070-mdarchecklist1.pdf]

Materials Design Analysis Reporting (MDAR)

Checklist for Authors

The MDAR framework establishes a minimum set of requirements in transparent reporting mainly applicable to studies in the life sciences.

eLife asks authors to **provide detailed information within their article** to facilitate the interpretation and replication of their work. Authors can also upload supporting materials to comply with relevant reporting guidelines for health-related research (see EQUATOR Network), life science research (see the BioSharing Information Resource), or animal research (see the ARRIVE Guidelines and the STRANGE Framework; for details, see *eLife*'s Journal Policies). Where applicable, authors should refer to any relevant reporting standards materials in this form.

For all that apply, please note **where in the article** the information is provided. Please note that we also collect information about data availability and ethics in the submission form.

Materials:

**Newly created materials**	**Indicate where provided: section/figure legend**	**N/A**
The manuscript includes a dedicated "materials availability statement" providing transparent disclosure about availability of newly created materials including details on how materials can be accessed and describing any restrictions on access.	**Newly generated plasmids:** Materials and Methods, "Stable organoid line generation" section. **CRISPR-engineered organoid lines:** Materials and Methods, "Stable organoid line generation" section.	

**Antibodies**	**Indicate where provided: section/figure legend**	**N/A**
For commercial reagents, provide supplier name, catalogue number and RRID, if available.	Materials and Methods, "Reagent Table, Antibodies"	

**DNA and RNA sequences**	**Indicate where provided: section/figure legend**	**N/A**

Short novel DNA or RNA including primers, probes: Sequences should be included or deposited in a public repository.	All primer and gRNA sequences are listed in Supplementary File 1C	

**Cell materials**	**Indicate where provided: section/figure legend**	**N/A**
Cell lines: Provide species information, strain. Provide accession number in repository OR supplier name, catalog number, clone number, OR RRID.	Materials and Methods, "Reagent Table, Cell lines"	
Primary cultures: Provide species, strain, sex of origin, genetic modification status.	Materials and Methods, "Mice" section	

**Experimental animals**	**Indicate where provided: section/figure legend**	**N/A**
Laboratory animals or Model organisms: Provide species, strain, sex, age, genetic modification status. Provide accession number in repository OR supplier name, catalog number, clone number, OR RRID.	Materials and Methods, "Mice" Section	
Animal observed in or captured from the field: Provide species, sex, and age where possible.		N/A

**Plants and microbes**	**Indicate where provided: section/figure legend**	**N/A**
Plants: provide species and strain, ecotype and cultivar where relevant, unique accession number if available, and source (including location for collected wild specimens).		N/A
Microbes: provide species and strain, unique accession number if available, and source.		N/A

**Human research participants**	**Indicate where provided: section/figure legend) or state if these demographics were not collected**	**N/A**

If collected and within the bounds of privacy constraints report on age, sex, gender and ethnicity for all study participants.		N/A

Design:

**Study protocol**	**Indicate where provided: section/figure legend**	**N/A**
If the study protocol has been pre-registered, provide DOI. For clinical trials, provide the trial registration number OR cite DOI.		N/A

**Laboratory protocol**	**Indicate where provided: section/figure legend**	**N/A**
Provide DOI OR other citation details if detailed step-by-step protocols are available.		N/A

**Experimental study design (statistics details) ***		
**For in vivo studies: State whether and how the following have been done**	**Indicate where provided: section/figure legend. If it could have been done, but was not, write "not done"**	**N/A**
Sample size determination	Sample sizes are indicated in the Results, Figures and Figure legends. See Figure 3 and Figure 6. Sample sizes were determined based on preliminary experiments and prior experience.	
Randomisation	Animals were randomly assigned to experimental groups.	
Blinding	Tumor measurements and histological/IHC analyses were performed blinded to experimental group.	
Inclusion/exclusion criteria	Tumor outgrowth criteria and humane endpoint criteria are described in Materials and	

	Methods, “In vivo transplantation and tumor dissection.”	

**Sample definition and in-laboratory replication**	**Indicate where provided: section/figure legend**	**N/A**
State number of times the experiment was replicated in the laboratory.	Experiments were independently replicated as indicated in the corresponding figure legends, Materials and Methods and Table S5.	
Define whether data describe technical or biological replicates.	Data represent biological replicates unless otherwise specified; technical replicates were not treated as independent biological replicates.	

**Ethics**	**Indicate where provided: section/submission form**	**N/A**
Studies involving human participants: State details of authority granting ethics approval (IRB or equivalent committee(s), provide reference number for approval.		N/A
Studies involving experimental animals: State details of authority granting ethics approval (IRB or equivalent committee(s), provide reference number for approval.	All animal experiments were approved by the Institutional Animal Care and Use Committee of Southern University of Science and Technology (SUSTech) (approval number: SUSTech-JY202401002).	
Studies involving specimen and field samples: State if relevant permits obtained, provide details of authority approving study; if none were required, explain why.		N/A

**Dual Use Research of Concern (DURC)**	**Indicate where provided: section/submission form**	**N/A**

If study is subject to dual use research of concern regulations, state the authority granting approval and reference number for the regulatory approval.		N/A

Analysis:

**Attrition**	**Indicate where provided: section/figure legend**	**N/A**
Describe whether exclusion criteria were pre-established. Report if sample or data points were omitted from analysis. If yes, report if this was due to attrition or intentional exclusion and provide justification.	Exclusion criteria were pre-established as described in the Methods. No samples or data points were excluded from the reported analyses.	

**Statistics**	**Indicate where provided: section/figure legend**	**N/A**
Describe statistical tests used and justify choice of tests.	Statistical tests were selected according to the data type and experimental design and are specified in the corresponding figure legends and Materials and Methods, "Statistical analysis" section.	

**Data availability**	**Indicate where provided: section/submission form**	**N/A**
For newly created and reused datasets, the manuscript includes a data availability statement that provides details for access (or notes restrictions on access).	The manuscript includes a Data Availability Statement describing access to the newly generated bulk RNA-seq datasets, and a reused dataset for analysis as described in Materials and Methods, "ssGSEA of clinical samples" section.	
When newly created datasets are publicly available, provide accession number in repository OR DOI and licensing details where available.	Newly generated bulk RNA-seq data are publicly available in the NCBI Gene Expression Omnibus (GEO) under accession number	

	GSE300119.	
If reused data is publicly available provide accession number in repository OR DOI, OR URL, OR citation.	The gene expression data for the CRC cohort were obtained from the sequencing data by laser-capture microdissection, which was deposited in Zenodo with accession number: 10927005. DOI: 10.1016/j.ccell.2024.12.008	

**Code availability**	**Indicate where provided: section/figure legend**	**N/A**
For any computer code/software/mathematical algorithms essential for replicating the main findings of the study, whether newly generated or re-used, the manuscript includes a data availability statement that provides details for access or notes restrictions.	All computational analyses were performed using previously published software and methods as described and cited in the Materials and Methods, “RNA-seq analysis” section and References. No custom software or code essential for reproducing the main findings was generated for this study.	
Where newly generated code is publicly available, provide accession number in repository, OR DOI OR URL and licensing details where available. State any restrictions on code availability or accessibility.		N/A
If reused code is publicly available provide accession number in repository OR DOI OR URL, OR citation.	Previously published software and R packages used in this study are cited in the Materials and Methods, “RNA-seq analysis” section and References.	

Reporting:

The MDAR framework recommends adoption of discipline-specific guidelines, established and endorsed through community initiatives.

**Adherence to community standards**	**Indicate where provided: section/figure legend**	**N/A**

State if relevant guidelines (e.g., ICMJE, MIBBI, ARRIVE, STRANGE) have been followed, and whether a checklist (e.g., CONSORT, PRISMA, ARRIVE) is provided with the manuscript.	Relevant guidelines were followed, including the ARRIVE 2.0 guidelines for reporting animal experiments. No separate ARRIVE checklist was provided with the manuscript.	

---

* We provide the following guidance regarding transparent reporting and statistics; we also refer authors to Ten common statistical mistakes to watch out for when writing or reviewing a manuscript.

Sample-size estimation

- You should state whether an appropriate sample size was computed when the study was being designed
- You should state the statistical method of sample size computation and any required assumptions
- If no explicit power analysis was used, you should describe how you decided what sample (replicate) size (number) to use

Replicates

- You should report how often each experiment was performed
- You should include a definition of biological versus technical replication
- The data obtained should be provided and sufficient information should be provided to indicate the number of independent biological and/or technical replicates
- If you encountered any outliers, you should describe how these were handled
- Criteria for exclusion/inclusion of data should be clearly stated
- High-throughput sequence data should be uploaded before submission, with a private link for reviewers provided (these are available from both GEO and ArrayExpress)

Statistical reporting

- Statistical analysis methods should be described and justified
- Raw data should be presented in figures whenever informative to do so (typically when N per group is less than 10)
- For each experiment, you should identify the statistical tests used, exact values of N, definitions of center, methods of multiple test correction, and dispersion and precision measures (e.g., mean, median, SD, SEM, confidence intervals; and, for the major substantive results, a measure of effect size (e.g., Pearson's r, Cohen's d)
- Report exact p-values wherever possible alongside the summary statistics and 95% confidence intervals. These should be reported for all key questions and not only when the p-value is less than 0.05.

Group allocation

- Indicate how samples were allocated into experimental groups (in the case of clinical studies, please specify allocation to treatment method); if randomization was used, please also state if restricted randomization was applied
- Indicate if masking was used during group allocation, data collection and/or data analysis
