# Supplementary material for: Impacts of mutation accumulation and order on tumor initiation revealed by engineered murine colorectal cancer organoids
Source: eLife. 2026 Nov 3;15:RP110070. doi: 10.7554/eLife.110070 (PMC13730527; doi:10.7554/eLife.110070)
Supplement: Supplementary file 2 [file elife-110070-fig1-figsupp1-data1.zip › Figure 1ΓÇôfigure supplement 1ΓÇôSource data 1.pdf]

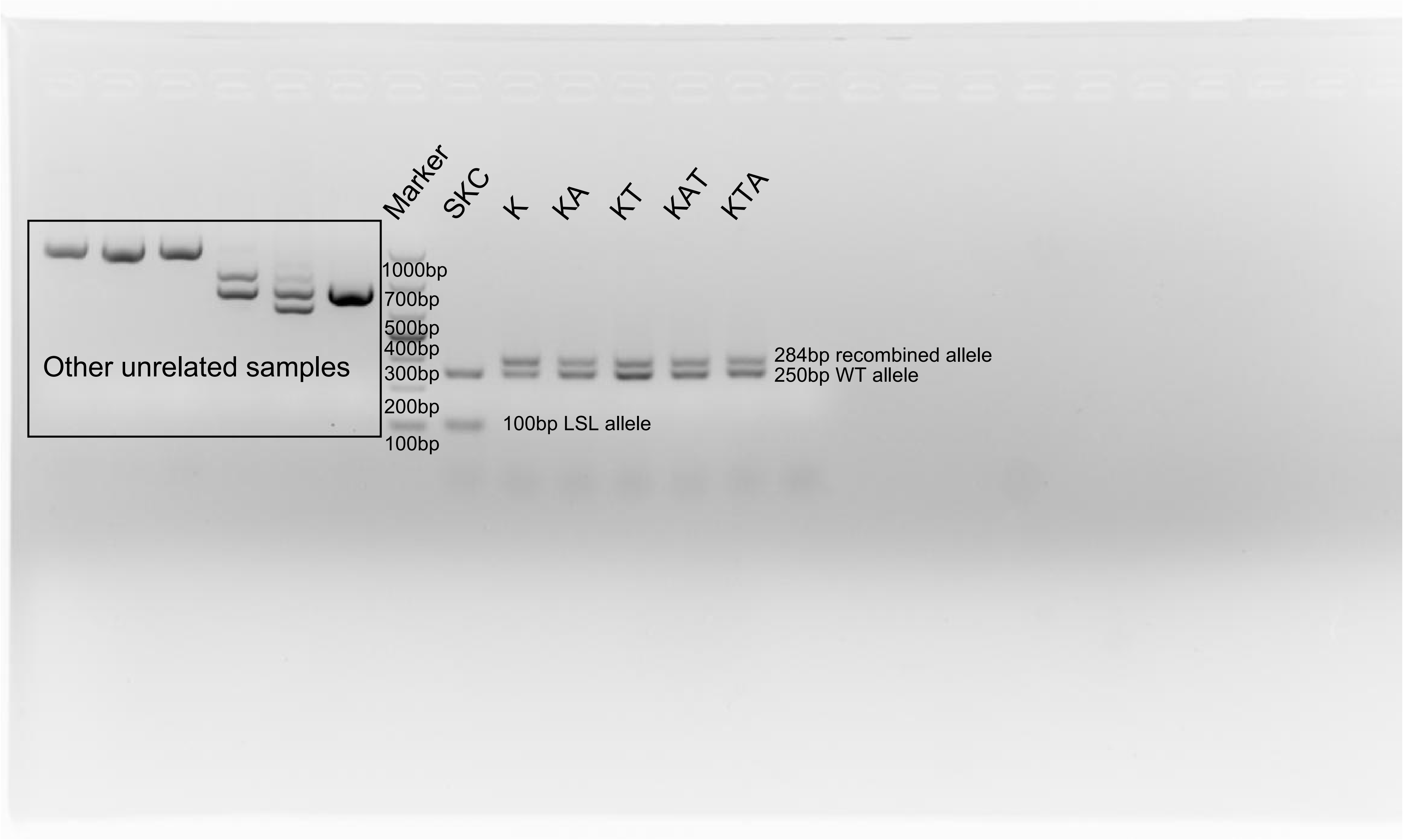

Marker
SKC
K
KA
KT
KAT
KTA
Other unrelated samples
1000bp
700bp
500bp
400bp
300bp
200bp
100bp
100bp LSL allele
284bp recombined allele
250bp WT allele
