# Supplementary material for: Impacts of mutation accumulation and order on tumor initiation revealed by engineered murine colorectal cancer organoids
Source: eLife. 2026 Nov 3;15:RP110070. doi: 10.7554/eLife.110070 (PMC13730527; doi:10.7554/eLife.110070)
Supplement: Peer review history

# Editor report: eLife Assessment

**DOI:** 10.7554/eLife.110070.3.sa0

**Authors:** Yongliang Yang

**Affiliations:** Shanghai University of Medicine and Health Sciences China

**Keywords:** Solid, Valuable

This study presents a **valuable** finding on impacts of the common driver mutations APC, KRAS G12D, and TP53 in murine colorectal organoids. In particular, the authors examine how the order of APC and TP53 acquisition influences tumor phenotype. The evidence supporting the claims of the authors is **solid**. The work will be of interest to scientists working in the field of coloreatal cancer.

# Referee report: Reviewer #2 (Public review)

**DOI:** 10.7554/eLife.110070.3.sa1

Summary:

This study addresses an important and timely question in colorectal cancer biology by systematically examining the effects of the common driver mutations APC, KRAS G12D, and TP53 in murine colorectal organoids, with particular emphasis on how the order of APC and TP53 acquisition influences tumor phenotype. These mutations are well known to be frequent, truncal, and often co-occurring in colorectal cancer. While it is increasingly appreciated that mutational order can shape tumor behavior, studies directly comparing the phenotypic consequences of alternative APC-TP53 mutation orders remain rare. This work therefore addresses a relevant and timely question.

Strengths:

A major strength of the study is its focus on previously unexplored biology, combined with the generation of multiple isogenic murine organoid models with controlled mutational sequences. The authors employ careful and robust quality control of the CRISPR-mediated alterations, and the inclusion of both in vitro and in vivo experiments strengthens the relevance of the work.

Weaknesses:

There are, however, several limitations that should be considered when interpreting the findings. First, KRAS G12D activation is used as the initiating alteration, whereas APC loss is generally believed to be the initiating event in most human colorectal cancers. Second, the analysis is restricted to comparing only two mutation orders (KAT versus KTA), which limits the breadth of conclusions that can be drawn about mutation ordering more generally. Finally, key RNA-sequencing and in vivo experiments rely on a limited number of isogenic lines, which constrains interpretability.

The study aimed to systematically investigate how the accumulation and sequence of driver mutations influence colorectal cancer initiation. The data provide intriguing evidence that the relative timing of APC and TP53 loss may impact tumor initiation and survival in a hostile microenvironment. However, given the limited number of biological replicates, these observations should be interpreted with caution and would benefit from further validation.

# Author comment: Author response

**DOI:** 10.7554/eLife.110070.3.sa2

**Authors:** Yanping Li, Xiaoxin Xie, Daqi Deng, Zhiyuan Sun, Zhaoan Huang, Yisen Tang, Liang Fang, Wei Chen, Qionghua Zhu

**Affiliations:** Southern University of Science and Technology Shenzhen China; Southern University of Science and Technology Shenzhen China; Southern University of Science and Technology Shenzhen China; Southern University of Science and Technology Shenzhen China; Southern University of Science and Technology Shenzhen China; Southern University of Science and Technology Shenzhen China; Southern University of Science and Technology Shenzhen China; Southern University of Science and Technology Shenzhen China; Southern University of Science and Technology Shenzhen China

The following is the authors’ response to the original reviews.

**Public Reviews:**

**Reviewer #1 (Public review):**

Summary:

In this study, Li et al. used genetically engineered murine intestinal organoids to investigate how the temporal order of oncogenic mutations influences cell state and tumourigenicity of colorectal epithelial cells. By sequentially introducing Apc and Trp53 loss-of-function mutations in alternate orders within a Kras^G12D background, the authors generated isogenic organoid lines for both in vitro and in vivo characterisation. Bulk RNA-seq reveals expected transcriptional changes with relatively modest differences between the two triple-mutant configurations (KAT vs KTA). The key finding emerges from transplantation assays: while KAT and KTA organoids show equivalent tumourigenic potential in immunodeficient mice, only KAT organoids form tumours in immunocompetent hosts (5/10 vs 0/10), suggesting that mutation order shapes susceptibility to immune-mediated clearance. The experiments are well-executed, and the conclusions are generally supported by the data.

Strengths:

The experimental system is well-designed for the question. By combining a Kras^G12D transgenic background with sequential CRISPR-mediated knockout of Apc and Trp53 in alternate orders, the authors generated truly isogenic organoid lines that differ only in mutational sequence. This is technically non-trivial and provides a clean platform for dissecting order effects, a question otherwise difficult to address experimentally.

The authors performed comprehensive baseline characterisation of these organoids, including morphological and histological assessment, quantification of organoid-forming efficiency and proliferation, and bulk RNA-seq profiling. While these analyses revealed no major differences between KAT and KTA organoids, and the observed enhancement of epithelial stemness upon Apc loss and proliferative advantage conferred by Trp53 loss are largely expected, the systematic nature of this characterisation establishes a useful methodological template for future organoid-based studies.

The authors further investigated the functional impact of mutational order using subcutaneous transplantation assays. By comparing tumour formation in immunodeficient versus immunocompetent hosts, the authors uncover a genuinely unexpected finding: KAT and KTA organoids behave equivalently in the absence of adaptive immunity, but diverge dramatically when immune pressure is applied (KAT: 5/10; KTA: 0/10). This observation is arguably the most compelling aspect of the study and opens an interesting line of inquiry.

We greatly appreciate your comments on this study.

Weaknesses:

The authors acknowledge that initiating with Kras^G12D does not reflect the typical human sporadic CRC trajectory, where APC loss is usually the first event. While this design choice was pragmatic, it means the observed order effects are contextualised within an artificial starting point. It remains unclear whether the Apc/Trp53 order would matter in a Kras-wild-type background, or whether the Kras-driven cellular state is a prerequisite for these phenotypes to emerge.

We agree with the reviewer that initiating tumorigenesis with *Kras^G12D^* does not fully recapitulate the most common trajectory of sporadic human CRC, where APC loss typically occurs first. We had noted this point in the original Discussion and further clarified it more explicitly in the Introduction part of the revised manuscript as shown in Line 97–103.

Our experimental design was intended to establish a controlled and genetically tractable system to interrogate the principle of mutation order effects. In this context, *Kras^G12D^* activation provides a stable oncogenic baseline that facilitates sequential genome engineering and comparison of isogenic lines.

Although APC loss is frequently the initiation event, a recent study has suggested that *Kras^G12D^* priming can reshape the selective landscape for subsequent driver events, including *Apc* alterations (PMID: 41339549). Consistent with this notion, our data indicate that *Kras^G12D^* activation induces a permissive oncogenic cellular state that may influence the phenotypic consequences of later mutations. We therefore speculate that the *Kras^G12D^*-primed context may contribute to the observed order-dependent effects.

We agree that testing *Apc*/*Trp53* order in a *Kras*-wild-type background would be an important future direction, and we have pointed this out explicitly in the revised Discussion as shown in Line 549–554.

Subcutaneous implantation provides a tractable readout of tumourigenicity, but the cutaneous immune microenvironment differs substantially from that of the intestinal mucosa. Given that the central claim concerns immune-mediated selection, orthotopic transplantation would more directly test whether the observed order effects hold in a physiologically relevant context.

In the present study, we employed subcutaneous transplantation as a widely used platform to assess tumorigenic potential under controlled immune conditions. This approach offers high reproducibility, straightforward tumour monitoring, and has been broadly applied in organoid-based cancer studies in both immunodeficient (PMID: 23273993, 23776211, 32209571, 33055221) and immunocompetent (PMID: 32209571, 33055221, 41672595) settings.

Importantly, our primary goal was to determine whether mutation order influences susceptibility to immune-mediated clearance, rather than to model the full complexity of the intestinal niche. The clear divergence between KAT and KTA specifically in immunocompetent hosts supports the existence of intrinsic mutation order-dependent immune vulnerability.

Nevertheless, we fully agree with the reviewer that orthotopic transplantation would provide a more physiologically relevant immune microenvironment and represents also an important direction for future investigation. We have explicitly discussed this limitation and highlight orthotopic validation as an important future direction in the revised Discussion as shown in Line 563–571.

The ssGSEA comparison involves only 14 ATK tumours, and the key comparisons (Figure 6E) yield borderline significance (p=0.052). More fundamentally, since mutation order cannot be inferred from the clinical samples, the authors are correlating organoid-derived IFN signatures with tumour immunophenotypes without direct evidence that these patients' tumours followed a KAT-like trajectory. The reasoning becomes circular: KAT organoids define the signature used to identify KAT-like clinical tumours.

We thank the reviewer for raising this important point. We would like to clarify that our intention was not to infer the actual mutation order in clinical samples, which indeed cannot be reliably reconstructed from bulk tumour RNA-seq data.

Instead, our goal was to determine whether the transcriptional programs distinguishing KAT and KTA organoids could be observed in human CRC cohorts. In this context, the organoid-derived IFN-related signature was used as a molecular reference to assess potential clinical correlation, rather than to classify tumours by evolutionary trajectory.

We agree that the statistical significance in Figure 6E is modest (p = 0.052), and we have revised the text (Line 478–480) to present this analysis more cautiously as a suggestive trend rather than definitive evidence. We also clarified this limitation explicitly in the revised manuscript (Line 537–542) to avoid overinterpretation.

Furthermore, the most striking finding of the study, that KTA organoids fail to form tumours in immunocompetent hosts while KAT organoids can, lacks a mechanistic follow-up. The transcriptomic differences between KAT and KTA are modest when cultured as monocultures, yet their in vivo fates diverge dramatically. The authors do not address why these subtle intrinsic differences translate into such divergent immune susceptibility, nor do they characterise the immune response adequately (beyond limited CD4/CD8 IHC at tumour peripheries).

We thank the reviewer for this important point. We agree that the mechanistic basis underlying the differential immune susceptibility between KAT and KTA remains incompletely resolved.

A practical limitation of the current study is that KTA grafts failed to establish tumours in immunocompetent hosts, which precluded downstream histological and immune profiling of established lesions. As a result, our in vivo immune characterization of KTA grafts is nearly impossible.

Nevertheless, our transcriptomic analyses indicate that KAT and KTA organoids differ in interferon-response and immune-related programs prior to transplantation, and those differentially expressed genes were consistently preserved in tumour cells derived from immunodeficient hosts. These results suggest the presence of intrinsic tumour-cell-autonomous differences may influence immune recognition or clearance.

We have expanded the Discussion to outline several non-mutually exclusive mechanisms that could account for this phenotype, including altered interferon responsiveness, differential antigen presentation capacity, and changes in tumour cell-intrinsic immune escape programs (Line 527–533). These hypotheses are consistent with the transcriptional differences observed prior to transplantation and provide a framework for future mechanistic investigation. We agree that deeper immune profiling (e.g., immune infiltrate composition, antigen presentation status, and functional immune assays) will be important to fully elucidate the mechanism and represents a key direction for future work.

**Reviewer #2 (Public review):**

Summary:

This study addresses an important and timely question in colorectal cancer biology by systematically examining the effects of the common driver mutations APC, KRAS G12D, and TP53 in murine colorectal organoids, with particular emphasis on how the order of APC and TP53 acquisition influences tumor phenotype. These mutations are well known to be frequent, truncal, and often co-occurring in colorectal cancer. While it is increasingly appreciated that mutational order can shape tumor behavior, studies directly comparing the phenotypic consequences of alternative APC-TP53 mutation orders remain rare. This work, therefore, addresses a relevant and timely question.

Strengths:

A major strength of the study is its focus on previously unexplored biology, combined with the generation of multiple isogenic murine organoid models with controlled mutational sequences. The authors employ careful and robust quality control of the CRISPR-mediated alterations, and the inclusion of both in vitro and in vivo experiments strengthens the relevance of the work.

We greatly appreciate your comments on this study.

Weaknesses:

There are, however, several limitations that should be considered when interpreting the findings. First, KRAS G12D activation is used as the initiating alteration, whereas APC loss is generally believed to be the initiating event in most human colorectal cancers.

We sincerely thank the reviewer for their insightful comments regarding the initiation of tumorigenesis with a *Kras* mutation rather than the more canonical *Apc* loss, which was also raised by the reviewer #1. We fully agree that the *Apc*-first represents the most prevalent sequence in human colorectal cancer (CRC), We have more clearly explained the rationale for our experimental design in the revised Introduction part as outlined in our response to reviewer #1.

Second, the analysis is restricted to comparing only two mutation orders (KAT versus KTA), which limits the breadth of conclusions that can be drawn about mutation ordering more generally.

We thank the reviewer for this critical concern, which we agree is essential for strengthening the robustness and generality of our findings. However, as a proof-of-concept study of *Apc* and *Trp53* loss, two major oncogenic events in CRC, serves as a biologically meaningful starting point for dissecting order-dependent effects. Although it is of great significance to compare all six possible mutation orders of these three driver genes, generating and thoroughly characterizing all genotypes (with identical replicates) represents a substantial undertaking beyond the scope of this initial study.

Finally, key RNA-sequencing and in vivo experiments rely on a single isogenic line, which substantially constrains interpretability.

The aim of the study was to systematically investigate how mutation accumulation and order influence colorectal cancer initiation. While the data suggest that the relative timing of APC and TP53 loss may be particularly important for tumor initiation, the absence of biological replication makes it difficult to draw robust conclusions. Engraftment efficiency and tumor behavior can be influenced by many factors for a single clone, including additional passenger mutations acquired during culturing, as well as epigenetic differences that are independent of the engineered mutations.

We thank the reviewer for this concern. We apologize that we have not made a clear presentation of our data source. Indeed, for all major in vitro and in vivo assays of double and triple mutants, we analyzed at least two independently derived clones per genotype. These independent clones harbour distinct mutations in target genes and were treated as biological replicates throughout the study.

To improve clarity and transparency, we have revised the relevant figure legends and further provided a Supplementary file 1E to explicitly indicate the clonal origin of each data point throughout the study.
